# Clinical and Histopathological Predictors of Recurrence in Uterine Smooth Muscle Tumor of Uncertain Malignant Potential (STUMP): A Multicenter Retrospective Cohort Study of Tertiary Centers

**DOI:** 10.1245/s10434-022-12353-y

**Published:** 2022-08-17

**Authors:** Fulvio Borella, Stefano Cosma, Domenico Ferraioli, Isabelle Ray-Coquard, Nicolas Chopin, Pierre Meeus, Vincent Cockenpot, Giorgio Valabrega, Giulia Scotto, Margherita Turinetto, Nicoletta Biglia, Luca Fuso, Luca Mariani, Dorella Franchi, Ailyn Mariela Vidal Urbinati, Ida Pino, Gianluca Bertschy, Mario Preti, Chiara Benedetto, Isabella Castellano, Paola Cassoni, Luca Bertero

**Affiliations:** 1 Division of Gynecology and Obstetrics 1, Department of Surgical Sciences, City of Health and Science University Hospital, University of Turin, Turin, Italy; 2 Léon Bérard Comprehensive Cancer Center, Lyon, France; 3 Department of Oncology, University of Turin, Turin, Italy; 4 Division of Obstetrics and Gynecology - A.O. Ordine Mauriziano, University of Turin, Turin, Italy; 5 Preventive Gynecology Unit, European Institute of Oncology IRCCS, Milan, Italy; 6 Pathology Unit, Department of Medical Sciences, University and City of Health and Science, Turin, Italy

## Abstract

**Background:**

The term uterine smooth muscle tumor of uncertain malignant potential (STUMP) indicates a rare, equivocal entity between benign leiomyomas and leiomyosarcomas. In the present study, we evaluated a comprehensive range of clinical, surgical, and pathological features in a large multicenter series of patients with STUMP to identify risk factors for recurrence.

**Methods:**

This is a retrospective study performed by collecting consecutive cases diagnosed between January 2000 and December 2020 in five tertiary centers. Associations between STUMP recurrence and clinicopathological characteristics as well as surgical treatment modality were investigated.

**Results:**

Eighty-seven patients affected by STUMP were considered. Of them, 18 cases (20.7%) recurred: 11 as leiomyosarcoma (LMS) and 7 as STUMP. The mean time to recurrence was 79 months. We found that fragmentation/morcellation, epithelioid features, high mitotic count, Ki-67 value > 20%, progesterone receptor (PR) < 83%, and p16 diffuse expression were associated with higher risk of recurrence and shorter recurrence-free survival (RFS). Furthermore, morcellation/fragmentation and mitotic count remained independent risk factors for recurrence and shorter RFS after multivariate analysis, while the presence of epithelioid features was an independent risk factor for recurrence only.

**Conclusions:**

Our results suggest that morcellation is associated with risk of recurrence and shorter RFS, thus it should be avoided if a STUMP is suspected preoperatively. Epithelioid features, high proliferation activity, low PR expression, and diffuse p16 expression are also unfavorable prognostic factors, so patients presenting these features should be closely followed up.

Uterine smooth muscle tumors of uncertain malignant potential (STUMPs) are extremely rare neoplasms, and available literature is limited to small and mainly monocentric retrospective series.^1–12^ In 1994, Bell et al.^13^ reviewed 213 problematic uterine smooth muscle tumors (USMTs) proposing STUMP diagnostic criteria on the basis of the presence of tumor cell atypia, necrosis, and mitotic figures [assessed as to their count per 10 high-power fields (HPFs)]. In the following years, the World Health Organization (WHO) Classification of Tumors of Female Reproductive Organs defined uterine STUMP as a USMT that cannot be diagnosed unequivocally as benign or malignant and does not satisfy all the diagnostic criteria for a leiomyosarcoma (LMS).^14^

Some authors have also tried to identify useful tools for the preoperative diagnosis of STUMPs, but to date, none has entered clinical practice.^6,15^

Furthermore, there is no consensus about the postoperative management of these tumors. Despite these uncertainties, protocols for the management of these neoplasms would be highly warranted since recurrence is relatively frequent. Indeed, a recent review reported an overall recurrence rate of 11–13% as either STUMP or LMS,^16^ but other authors reported even higher recurrence rates, up to 36.4% in a monocentric series of 22 STUMPs.^8^

Identification of prognostic markers of recurrence is warranted since to date, there are no validated clinical or pathological parameters which predict the longitudinal behavior of these lesions.

The aim of this study is thus to evaluate a wide range of clinical, surgical, and pathological characteristics in a large, multicenter, patient series to identify risk factors of recurrence that could be exploited to tailor the management of these rare neoplasms.

## Materials and Methods

A multicenter, retrospective, cohort study was performed collecting consecutive cases diagnosed between January 2000 and December 2020 in five tertiary centers (A.O.U. Città della Salute e della Scienza, S. Anna Hospital, Turin, Italy; Mauriziano Hospital, Turin, Italy; Candiolo Cancer Institute, FPO-IRCCS, Candiolo, Italy; European Institute of Oncology IRCCS, Milan, Italy; Leon Berard Cancer Center, Lyon, France). We searched the archives of the Gynecological Oncology and Pathology units of each institution for all cases classified as STUMP. All samples were reviewed by two expert pathologists (I.C., L.B.) to confirm the diagnostic assessment of STUMP according to the most recent diagnostic criteria provided by the 2020 WHO Classification of Tumors of Female Reproductive Organs^14^ [i.e., USMTs not fitting the diagnostic criteria of leiomyoma or LMS: (a) absent necrosis, focal/multifocal/diffuse moderate to severe atypia, < 10 mitoses/10 HPFs; (b) presence of necrosis, none or mild atypia, < 10 mitoses/10 HPFs; and (c) absent necrosis, none atypia, > 15 mitoses/10 HPFs]. Conversely, LMS diagnosis requires the presence of two out of the three following features: marked cellular atypia, > 10 mitoses/10 HPFs, or necrosis.^14^

The following histopathological parameters were also recorded: cellularity, grade/extension of cellular atypia, highest mitotic count per 10 HPFs, presence of atypical mitoses, presence and type of necrosis (ischemic or coagulative), presence of myxoid, epithelioid or degenerative features, apoptosis and intravascular intrusions. Immunohistochemistry stains for Ki-67, estrogen receptor (ER), progesterone receptor (PR), p16, and p53 were performed whenever possible and assessed considering the rate of positive cells.

For outcome analyses, we considered as cutoff values the mean number of mitoses (per 10 HPFs), Ki-67 proliferation index (%), and EmiR and PR positive cells (%). We also tested the prognostic significance of cutoff values of 10 mitoses/10 HPFs, and 20% Ki-67 positivity, as some authors have reported their unfavorable prognostic significance in UMSTs.^10,17^

Patient data were extracted from medical records. The variables analyzed included age at diagnosis, number of previous pregnancies, menopausal status, body mass index (BMI), smoking status, symptoms, surgical procedure, largest tumor diameter (mm), and presence of associated leiomyomas and/or adenomyosis in surgically resected specimens. On the basis of pathological and surgical reports, we defined fragmentation as the manual/instrumental partial fragmentation of the neoplasm within the pelvic cavity during the enucleation, and morcellation as the division and removal of the lesion in small pieces through an abdominal incision or by vaginal route. No cases of in-bag morcellation were reported.

Disease recurrence was defined as any histologically confirmed relapse as STUMP or LMS. Survival time was measured from date of surgery until last follow-up, recurrence, or death from any cause.

Owing to the retrospective nature of the study and the lack of a standard follow-up protocol for STUMPs, differences were observed among centers/patients; however, in most cases, patients underwent a gynecological and ultrasound examination every 6 months for the first 5 years after initial diagnosis and then annually. In case of suspicion of recurrence, further diagnostic examinations were performed (abdominal/chest computed tomography, and/or positron emission tomography as appropriate).

Patients with no histological confirmation at pathological review or with follow-up shorter than 6 months were excluded.

Written consent was not required considering the retrospective nature of the study. The study was conducted in accordance with the Declaration of Helsinki and was approved by our local ethical committee (protocol number 0119045).

Statistical analyses were performed using IBM SPSS version 23 (SPSS Inc., Chicago, IL) software. Differences in proportions among categorical data of patients who had a recurrence and those who had not were assessed using Pearson’s chi-squared test or Fisher’s exact test. For continuous variables, the Shapiro–Wilk test was used to test data normality, and then, the Mann–Whitney *U*-test was used for data comparison. Survival times were analyzed using Kaplan–Meier curves and comparisons were performed by the log-rank test. A binary logistic regression model was performed using recurrence as the dependent variable and patient/STUMP characteristics as covariates. Multivariable logistic regression models were created by a backward stepwise procedure. The prognostic value of the variables was tested by univariate and multivariate analysis with the Cox regression model. Statistically significant values from Cox univariate analyses were entered into a multivariate analysis using the backward stepwise Cox regression model. All the analyses were conducted with a 95% confidence interval (CI), and a two-sided *p*-value of 0.05 was considered statistically significant.

## Results

### Clinical Characteristics

A total of 103 patients were initially considered. Sixteen patients with no histological confirmation at pathological review (*n* =12, 7 reclassified as a variant of leiomyoma, 5 as LMS) or with follow-up shorter than 6 months (*n* = 4) were excluded. Finally, 87 patients with a confirmed diagnosis of STUMP were included in the study (Table 1).

**Table 1:** Main demographics, clinical characteristics, and histological features of 87 STUMPs

	STUMP samples, *n* (%)
*Clinical characteristic*	
Ethnicity	
Caucasian	83 (95%)
Non-Caucasian	4 (5%)
Mean age (years) SD (range)	46 ± 10 (19–82)
Age (years)	
< 46	40 (46%)
≥ 46	47 (54%)
Number of pregnancies (NA 8)	
0	28 (35%)
≥ 1	51 (65%)
Menopause (NA 1)	
No	72 (84%)
Yes	14 (16%)
Mean BMI (SD, range)	25 ± 4 (19–40)
Smoking (NA 13)	
No	63 (85%)
Yes	11 (15%)
Abnormal uterine bleeding (NA 2)	
No	20 (24%)
Yes	65 (76%)
Pelvic pain (NA 2)	
No	62 (73%)
Yes	23 (27%)
Abdominal bloating (NA 2)	
No	69 (81%)
Yes	16 (19%)
Surgical procedure	
Hysterectomy	59 (68%)
Myomectomy	28 (32%)
Surgical approach	
Laparotomy	70 (80%)
Vaginal	4 (5%)
Laparoscopy	13 (15%)
Fragmentation/morcellation	
No	59 (68%)
Yes	28 (32%)
Surgical procedure (without fragmentation/morcellation)	
Hysterectomy	43 (73%)
Myomectomy	16 (27%)
Mean maximum diameter (mm) (NA = 7) SD (range)	73 ± 44 (5–230)
*Histological features*	
Associated leiomyomas (NA 1)	
No	40 (46%)
Yes	46 (54%)
Associated adenomyosis (NA 1)	
No	75 (87%)
Yes	11 (13%)
Atypia (severity)	
Absent/mild	59 (68%)
Moderate/severe	28 (32%)
Atypia (extension)	
Focal	60 (67%)
Diffuse	27 (33%)
Necrosis	
No	53 (61%)
Yes	34 (39%)
Ischemic	24 (71%)
Coagulative	10 (29%)
Hypercellularity	
No	45 (52%)
Yes	42 (48%)
Epithelioid features	
No	76 (87%)
Yes	11 (13%)
Myxoid features	
No	82 (94%)
Yes	5 (6%)
Degenerative features	
No	72 (83%)
Yes	15 (17%)
Atypical mitosis	
No	81 (93%)
Yes	6 (7%)
Apoptosis	
No	61 (70%)
Yes	26 (30%)
Vascular intrusion	
No	83 (95%)
Yes	4 (5%)
Mean number of mitoses/10 HPFs SD (range)	6 ± 7 (1–43)
Number of mitoses (according to the mean value)	
< 6	52 (60%)
≥ 6	35 (40%)
Number of mitoses (cutoff: 10/10 HPFs)	
≤ 10	74 (85%)
> 10	13 (15%)
Mean Ki-67 expression (%) SD (range) (NA = 10)	16 ± 15 (1–80)
Ki-67 (cutoff 16%) (according to the mean value) (NA = 10)	
≤ 16%	54 (70%)
> 16%	23 (30%)
Ki-67 (cutoff 20%) (NA = 10)	
≤ 20%	62 (80%)
> 20%	15 (20%)
Mean ER expression (%) SD (range) (NA = 21)	72 ± 20 (5–100)
ER expression (according to the mean value) (NA = 21)	
≥ 72%	34 (52%)
< 72%	32 (48%)
Mean PR expression (%) SD (range)	83 ± 18 (0–100)
PR expression (according to the mean value) (NA = 21)	
≥ 83%	47 (71%)
< 83%	19 (29%)
p53 expression (NA = 30)	
Negative	41 (72%)
Positive	16 (28%)
p16 expression (NA = 40)	
Absent/focal	28 (60%)
Widespread	19 (40%)

*BMI* body mass index, *ER* estrogen receptor, *NA* not available, *PR* progesterone receptor, *SD* standard deviation, *STUMP* smooth muscle tumors of uncertain malignant potential

The mean age at diagnosis was 46 (standard deviation (SD) 10, range 19–82) years, the most frequent ethnicity was Caucasian (83 of 87, 95% of patients), and the mean follow-up was 67 (SD ± 65, range 6–256) months.

The mean BMI was 25 (SD 4, range 19–40, 20 missing data), 51 of 79 patients (65%, 8 missing data) had previous pregnancies, 11 of 74 were smokers (15%, 13 missing data), and 65 of 85 (76%, 2 missing data) were symptomatic at the time of diagnosis.

Detailed data regarding the surgical procedures were collected. Most patients underwent hysterectomy (59 of 87, 68% of patients), while a laparoscopic approach was performed in only 12 of 87 (14%) patients.

In 28 of 87 (32%) cases, the tumor was morcellated or fragmented intraoperatively. In 12 of 28 (43%) patients who initially underwent a myomectomy, a hysterectomy was performed after the histological diagnosis of STUMP.

All STUMPs were confined to the uterine body at the time of first surgery, with a mean largest dimension of 73 (SD 44, range 5–230) mm.

### Histopathological Features

STUMP histopathological features were comprehensively analyzed. Moderate or severe cytological atypia was observed in 28 of 87 (68%) cases and diffuse cytological atypia in 27 of 87 (33%). Necrosis was observed in 34 of 87 (39%) cases: in 24 (71%) samples, the findings were consistent with ischemic necrosis, and in 10 (29%) with coagulative necrosis. High cellularity was observed in about half of the STUMPs (42 of 87, 48% of cases), and apoptotic figures in 26 of 87 (30%) cases. Other histopathological features (epithelioid or myxoid patterns, degenerative features, and the presence of atypical mitosis and/or vascular intrusions) were rarely present. The mean value of mitoses/10 HPFs was 6 (SD 7, range 1–43) and cases with a mitotic count/10 HPFs ≥ 6 and > 10 were 35 (40%) and 13 (15%), respectively.

The mean Ki-67 value (10 missing data) was 16% (SD 15%, range 1–80%), while a Ki-67 value > 20% was observed in 15 of 77 (20%) cases. Mean ER- and PR-positive cells were 72% (21 missing data, SD ± 20, range 5–100%) and 83% (21 missing data, SD 18%, range 0–100%), respectively. ER expression < 72% was observed in 32 of 66 (48%) cases, and PR expression < 83% in 19 of 66 (29%) STUMPs. Finally, p53 and p16 were found to be expressed in 16 of 57 (30 missing data, 28%) and 19 of 47 (40 missing data, 40%) cases.

Associated leiomyomas and/or adenomyosis were reported in 46 of 86 (1 missing data, 54%) and 11 of 86 (1 missing data, 13%) of the surgical specimens, respectively.

### Outcome Analysis

Overall, 18 (20.7%) cases recurred: 11 as LMS (12%) and 7 as STUMP (7.8%). The mean time to recurrence was 79 (SD 55, range 10–174) months. Of the 18 STUMPs that recurred, 5 (5.7%) patients died because of this disease [disease-specific survival (DSS): 94.3%].

No significant differences were observed between patients with and without disease recurrence in terms of age, number of previous pregnancies, BMI, type of symptoms, menopausal, and smoking status.

Although recurrence rates were not different according to the specific surgical procedure or approach, more recurrences were observed after intraoperative tumor fragmentation/morcellation (*p* = 0.003).

The following histopathological and immunohistochemical features showed a significantly different distribution according to recurrence: (a) presence of epithelioid features (*p* = 0.009), (b) higher mitotic count/10 HPFs (*p* = 0.01), (c) mitotic count ≥ 6/10 HPFs (mean value of mitotic count in the whole series) (*p* = 0.042) and > 10 per 10 HPFs (*p* = 0.004), (d) Ki-67 value > 20% (*p* = 0.04), (e) lower PR expression (*p* = 0.048), (f) PR expression < 83% (mean value of PR expression in the whole series) (*p* =0.036), and (g) diffuse p16 expression (*p* = 0.01).

Distribution of all clinical and histopathological characteristics according to recurrence is presented in Table 2.

**Table 2:** Distribution of the clinical and pathological features according to recurrence (in bold significant *p*-values)

	No recurrence (*n* = 69)	Recurrence (*n* = 18)	*p-*value
*Clinical characteristic*			
Ethnicity			
Caucasian	65 (78%)	18 (22%)	0.57
Non-Caucasian	4 (100%)	0 (0%)	
Mean age (years) SD (range)	46 ± 8 (29–77)	47 ± 16 (19–82)	0.49
Age (years)			
< 46	39 (83%)	8 (17%)	0.36
≥ 46	30 (75%)	10 (25%)	
Number of pregnancies (NA 8)			
0	20 (71%)	8 (29%)	0.11
≥ 1	44 (86%)	7 (14%)	
Menopause (NA 1)			
No	59 (82%)	13 (18%)	0.16
Yes	9 (64%)	5 (36%)	
Mean BMI SD (range)	25.4 ± 3.8 (19–40)	24 ± 6 (19–39)	0.49
Smoking (NA 13)			
No	52 (82%)	11 (18%)	0.21
Yes	7 (64%)	4 (36%)	
Abnormal uterine bleeding (NA 2)			
No	17 (85%)	3 (15%)	0.59
Yes	50 (77%)	15 (23%)	
Pelvic pain (NA 2)			
No	50 (81%)	12 (19%)	0.50
Yes	17 (74%)	6 (26%)	
Abdominal bloating (NA 2)			
No	54 (78%)	15 (22%)	1.00
Yes	13 (81%)	3 (19%)	
Surgical procedure			
Hysterectomy	46 (78%)	13 (22%)	0.78
Myomectomy	23 (82%)	5 (18%)	
Surgical approach			
Laparotomy	57 (82%)	13 (18%)	0.48
Vaginal	3 (75%)	1 (25%)	
Laparoscopy	9 (67%)	4 (33%)	
Fragmentation/morcellation			
No	52 (88%)	7 (12%)	**0**.**003**
Yes	17 (60%)	11 (40%)	
Surgical procedure (without fragmentation/morcellation)			
Hysterectomy	38 (88%)	5 (12%)	1.00
Myomectomy	14 (88%)	2 (12%)	
Mean maximum diameter (mm) (NA = 7) SD (range)	72.9 ± 45 (5–230)	73.8 ± 42 (20–150)	0.94
*Histological features*			
Associated leiomyomas (NA 1)			
No	31 (78%)	9 (22%)	0.74
Yes	37 (80%)	9 (20%)	
Associated adenomyosis (NA 1)			
No	58 (78%)	17 (22%)	0.44
Yes	10 (91%)	1 (9%)	
Atypia (severity)			
Absent/mild	47 (78%)	12 (22%)	0.87
Moderate/severe	22 (80%)	6 (20%)	
Atypia (extension)			
Focal	48 (80%)	12 (20%)	0.81
Diffuse	21 (78%)	6 (22%)	
Necrosis			
No	43 (81%)	10 (19%)	0.60
Yes	26 (76%)	8 (24%)	
Ischemic	20 (83%)	4 (17%)	0.19
Coagulative	6 (60%)	4 (40%)	
Hypercellularity			
No	38 (84%)	7 (16%)	0.22
Yes	31 (74%)	11 (26%)	
Epithelioid features			
No	64 (84%)	12 (16%)	**0.009**
Yes	5 (46%)	6 (54%)	
Myxoid features			
No	66 (80%)	16 (20%)	0.27
Yes	3 (60%)	2 (40%)	
Degenerative features			
No	57 (79%)	15 (21%)	1.00
Yes	12 (80%)	3 (20%)	
Atypical mitosis			
No	65 (80%)	16 (20%)	0.59
Yes	4 (67%)	2 (33%)	
Apoptosis			
No	49 (80%)	12 (20%)	0.72
Yes	20 (77%)	6 (23%)	
Vascular intrusion			
No	66 (80%)	17 (20%)	1.00
Yes	3 (75%)	1 (15%)	
Mean number of mitoses/10 HPFs SD (range)	4.5 ± 4 (1–18)	11 ± 11 (1–43)	**0.01**
Number of mitoses (according to the mean value)			
< 6	45 (86%)	7 (14%)	**0.042**
≥ 6	24 (69%)	11 (31%)	
Number of mitoses (cutoff 10/10 HPFs)			
≤ 10	61 (82%)	10 (18%)	**0.004**
> 10	8 (62%)	8 (38%)	
Mean Ki-67 expression (%) SD (range) (NA = 10)	13 ± 12 (1–70)	25 ± 23 (1–80)	**0.007**
Ki-67 (cutoff 20%) (NA = 10) (according to the mean value)			
≤ 16%	45 (83%)	9 (17%)	0.17
16%	16 (70%)	7 (30%)	
Ki-67 (cutoff 20%) (NA = 10)			
≤ 20%	52 (84%)	10 (16%)	**0.04**
> 20%	9 (60%)	6 (40%)	
Mean ER expression (%) SD (range) (NA = 21)	72 ± 18 (20–99)	69 ± 26 (5–100)	0.55
ER Expression (according to the mean value) (NA = 21)			
≥ 72%	26 (76%)	8 (24%)	0.98
< 72%	24 (75%)	8 (25%)	
Mean PR expression (%) SD (range)	85 ± 14 (30–100)	75 ± 26 (0–100)	**0.048**
PR expression (according to the mean value) (NA = 21)			
≥ 83%	39 (83%)	8 (17%)	**0.036**
< 83%	11 (58%)	8 (42%)	
p53 expression (NA = 30)			
Negative	35 (85%)	6 (15%)	0.44
Positive	12 (75%)	4 (25%)	
p16 expression (NA = 40)			
Absent/focal	27 (96%)	1 (4%)	**0.01**
Widespread	13 (68%)	6 (32%)	

*BMI* body mass index, *ER* estrogen receptor, *NA* not available, *PR* progesterone receptor, *SD* standard deviation, *STUMP* smooth muscle tumors of uncertain malignant potential

Logistic regression univariate analysis showed an association between multiple variables and recurrence (Table 3). Multivariate logistic regression showed that STUMP morcellation/fragmentation (OR 6.17, 95% CI 1.707–22.32, *p* = 0.006), mitotic count > 10/10 HPFs (OR 4.78, 95% CI 1.05–21.7, *p* = 0.043), and epithelioid features (OR 4.26, 95% CI 1.028–17.63, *p* = 0.046) were independent predictors of recurrence.

**Table 3:** Univariate analysis of clinicopathological variables associated with disease recurrence (in bold significant *p*-values)

Variable	Univariate analysis OR (95% CI)	*p*-value
Ethnicity		
Caucasian	1	0.43
Non-Caucasian	0.06 (0.00–1.43)	
Age (years)	1.018 (0.97–1.07)	0.49
Age < 45 years		
< 45	1	0.36
≥ 45	1.62 (0.57–4.61)	
Number of pregnancies		
0	1	0.11
≥ 1	0.39 (0.13–1.24)	
Menopause		
No	1	0.14
Yes	2.52 (0.72–8.77)	
BMI	0.94 (0.78–1.12)	0.49
Smoking		
No	1	0.61
Yes	2.70 (0.67–10.8)	
Abnormal uterine bleeding		
No	1	0.98
Yes	1.01 (0.36–2.88)	
Pelvic pain		
No	1	0.50
Yes	1.41 (0.48–4.52)	
Abdominal bloating		
No	1	0.79
Yes	0.83 (0.21–3.30)	
Surgical procedure		
Hysterectomy	1	0.77
Myomectomy	0.78 (0.24–2.42)	
Surgical approach		
Laparotomy	1	0.74
Vaginal	1.59 (0.43–5.81)	0.24
Laparoscopy	1.49 (0.14–15.46)	
Fragmentation/morcellation		
No	**1**	**0.005**
Yes	**4.80 (1.60–14.4)**	
Surgical procedure (without fragmentation/morcellation)		
Hysterectomy	1	0.92
Myomectomy	1.08 (0.18–6.25)	
Diameter (mm)	1.00 (0.99–1.01)	0.94
Associated leiomyomas		
No	1	0.74
Yes	0.83 (0.29–2.36)	
Associated adenomyosis		
No	1	0.34
Yes	0.34 (0.04–2.55)	
Cellular atypia		
Absent/mild	1	0.83
Moderate/severe	1.12 (0.38–3.25)	
Focal	1	0.81
Diffuse	1.14 (0.38–3.45)	
Necrosis		
No	1	0.60
Yes	1.32 (0.46–3.77)	
Ischemic	1	0.16
Coagulative	3.33 (0.63–17.5)	
Hypercellularity		
No	1	0.22
Yes	1.92 (0.67–5.55)	
Epithelioid features		
No	**1**	**0.007**
Yes	**6.4 (1.68–24.37)**	
Myxoid features		
No	1	0.29
Yes	2.75 (0.42–17.8)	
Degenerative features		
No	1	0.94
Yes	0.95 (0.24–3.80)	
Atypical mitoses		
No	1	0.44
Yes	2.03 (0.34–3.86)	
Apoptosis		
No	1	0.72
Yes	1.22 (0.40–3.71)	
Vascular intrusion		
No	1	0.82
Yes	1.29 (0.13–13.2)	
Number of mitoses/10 HPFs	**1.14 (1.04–1.24)**	**0.006**
Number of mitoses/10 HPFs		
< 6	**1**	**0.04**
≥ 6	**2.93 (1.01–8.58)**	
≤ 10	**1**	**0.03**
> 10	**2.94 (1.09–10.4)**	
Ki-67 expression	**1.04 (1.007–1.08)**	**0.017**
Ki-67 (cutoff 16%)		
< 16%	1	0.18
≥ 16%	2.19 (0.70–6.84)	
Ki-67 (cutoff 20%)		
≤ 20%	1	**0.048**
> 20%	**3.46 (1.008–11.9)**	
ER expression	0.99 (0.96–1.02)	0.55
ER expression (cutoff 72%)		
≥ 72%	1	0.89
< 72%	1.08 (0.35–3.34)	
PR expression	0.97 (0.94–1.002)	0.07
PR (cutoff 83%)		
≥ 83%	**1**	**0.04**
< 83%	**3.54 (1.08–11.6)**	
p53 expression		
Negative	1	0.36
Positive	1.94 (0.47–8.08)	
p16 expression		
Absent/focal	**1**	**0.03**
Widespread	**12.5 (1.35–114.5)**	

*BMI* body mass index, *ER* estrogen receptor, *NA* not available, *PR* progesterone receptor, *SD* standard deviation, *STUMP* smooth muscle tumors of uncertain malignant potential

Univariate Cox regression analysis (Table 4) showed that surgical fragmentation/morcellation (HR 3.68, 95% CI 1.42–9.54, *p* = 0.007), epithelioid features (HR 3.14, 95% CI 1.17–8.40, *p =* 0.022), higher mitotic count (HR 1.04, 95% CI 1.005–1.084, *p =* 0.03), presence of > 10 mitoses/10 HPFs (HR 2.84, 95% CI 1.10–5,19, *p =* 0.04), higher Ki-67 proliferation index (HR 1.033, 95% CI 1.009–1.057, *p* = 0.006), Ki67 > 20% (HR 3.06, 95% CI 1.07–8.73, *p* = 0.06), PR expression < 83% (HR 4.15, 95% CI 1.47–11.7, *p* = 0.007), and diffuse p16 expression (HR 13.1, 95% CI 1.56–11.7, *p* = 0.08) were associated with shorter RFS.

**Table 4:** Univariate analysis of the variables associated with time to STUMP recurrence (in bold significant *p*-values)

Variable	Univariate analysis HR (95% CI)	*p*-Value
Ethnicity		
Caucasian	1	0.43
Non-Caucasian	0.043 (0.00–5.43)	
Age (years)	1.017 (0.97–1.067)	0.49
Age < 45 years		
< 45	1	0.42
≥ 45	1.47 (0.57–3.77)	
Number of pregnancies		
0	1	0.217
≥ 1	0.53 (0.20–1.45)	
Menopause		
No	1	0.10
Yes	2.36 (0.84–6.67)	
BMI	0.95 (0.80–1.12)	0.55
Smoking		
No	1	0.10
Yes	2.57 (0.82–8.17)	
Abnormal uterine bleeding		
No	1	0.90
Yes	1.04 (0.41–2.64)	
Pelvic pain		
No	1	0.48
Yes	1.42 (0.53–3.79)	
Abdominal bloating		
No	1	0.82
Yes	1.15 (0.33–4.09)	
Surgical procedure		
Hysterectomy	1	0.92
Myomectomy	0.95 (0.34–2.70)	
Surgical approach		
Laparotomy	1	0.91
Vaginal	1.87 (0.20–16.7)	0.58
Laparoscopy	0.89 (0.12–6.88)	
Fragmentation/morcellation		
No	**1**	**0.007**
Yes	**3.68 (1.42–9.54)**	
Surgical procedure (without fragmentation/morcellation)		
Hysterectomy	1	0.89
Myomectomy	1.12 (0.2–5.8)	
Diameter (mm)	1.006 (0.99–1.02)	0.30
Associated leiomyomas		
No	1	0.77
Yes	0.87 (0.34–2.20)	
Associated adenomyosis		
No	1	0.30
Yes	0.30 (0.04–2.29)	
Cellular atypia		
Absent/mild	1	0.25
Moderate/severe	0.57 (0.21–1.50)	
Focal	1	0.83
Diffuse	1.11 (0.41–2.98)	
Necrosis		
No	1	0.40
Yes	1.49 (0.58–3.82)	
Ischemic	1	0.38
Coagulative	1.86 (0.46–7.54)	
Hypercellularity		
No	1	0.67
Yes	1.24 (0.47–3.23)	
Epithelioid features		
No	**1**	**0.022**
Yes	**3.14 (1.17–8.40)**	
Myxoid features		
No	1	0.64
Yes	1.42 (0.32–6.21)	
Degenerative features		
No	1	0.89
Yes	0.89 (0.25–3.07)	
Atypical mitoses		
No	1	0.20
Yes	2.59 (0.58–11.4)	
Apoptosis		
No	1	0.20
Yes	0.51 (0.17–1.44)	
Vascular intrusion		
No	1	0.63
Yes	1.60 (0.21–12.3)	
Number of mitoses/10 HPFs	**1.04 (1.005–1.084)**	**0.03**
Number of mitoses/10 HPFs		
< 6	1	0.07
≥ 6	2.40 (0.92–6.23)	
≤ 10	**1**	**0.04**
> 10	**2.84 (1.10–5.19)**	
Ki-67 expression	**1.033 (1.009–1.057)**	**0.006**
Ki-67 (cutoff 16%)		
< 16%	1	0.121
≥ 16%	2.23 (0.80–6.18)	
Ki-67 (cutoff 20%)		
≤ 20%	1	**0.036**
> 20%	**3.06 (1.07–8.73)**	
ER expression	0.998 (0.97–1.02)	0.80
ER expression (cutoff 72%)		
≥ 72%	1	0.56
< 72%	1.34 (0.50–3.61)	
PR expression	**0.97 (0.95–0.99)**	**0.003**
PR (cutoff 83%)		
≥ 83%	**1**	**0.007**
< 83%	**4.15 (1.47–11.7)**	
p53 expression		
Negative	1	0.10
Positive	3.20 (0.80–11.4)	
p16 expression		
Absent/focal	**1**	**0.018**
Widespread	**13.1 (1.56–111.7)**	

*BMI* body mass index, *CI* confidence interval; *ER* estrogen receptor, *HR* hazard ratio, *NA* not available, *PR* progesterone receptor, *SD* standard deviation, *STUMP* smooth muscle tumors of uncertain malignant potential

**Fig. 1:**
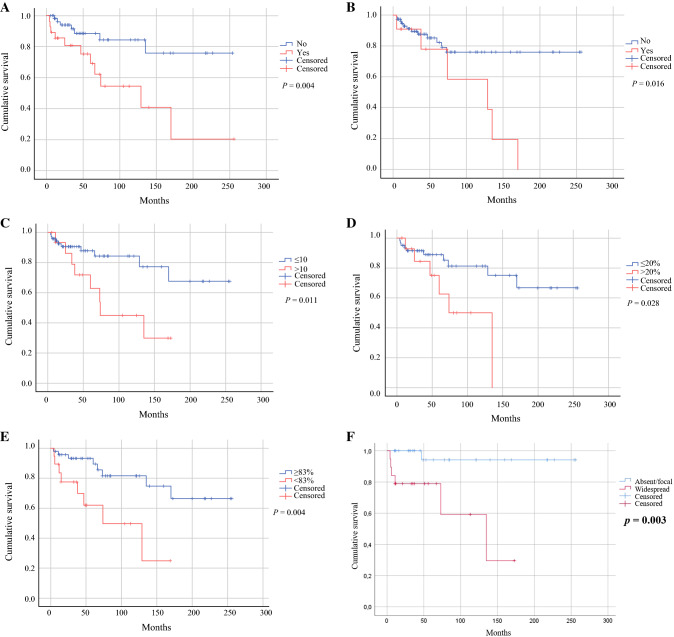
Kaplan–Meier curves for STUMP recurrence according to **A** morcellation, **B** epithelioid features, **C** number of mitoses > 10 per HPFs, **D** Ki-67 value > 20%, **E** PR value ≥ 83%, and **F** p16 expression

All the variables found to be significant in the univariate analysis also had an impact on recurrence-free survival (RFS) as calculated by the Kaplan–Meier method (Fig. 1).

By multivariate Cox analysis, morcellation/fragmentation (HR 5.65, 95% CI 1.53–20,8, *p* = 0.009) and mitotic count considered as a linear variable (HR 1.073, 95% CI 1.019–1.130, *p* = 0.008) were confirmed to be independently associated with RFS.

## Discussion

We comprehensively evaluated a wide range of clinical, surgical, and pathological features to ascertain their association with recurrence risk in STUMPs.

### Surgical Outcomes

Concerning clinical and surgical variables, the main finding of the present study is the association between morcellation/fragmentation and the risk of recurrence/shorter RFS. Overall, the optimal surgical approach to remove STUMP is a topic of debate given that there is no clear evidence whether surgical radicality offers a survival advantage; moreover, STUMPs can also occur during the reproductive age in patients desiring pregnancy, thus a conservative procedure must be taken into consideration.^16^ In our study, we found no differences in terms of recurrence rates according to surgical procedures, similar to the results reported by other authors.^2,8–11,18^ The same was not true for morcellation: recurrences were significantly more frequent after any type of morcellation (power or hand morcellation). This finding strongly supports the limited evidence available so far suggesting this association. In a small study on seven patients with a diagnosis of STUMP or endometrial stromal sarcoma who underwent power or hand morcellation during hysterectomy or myomectomy, six cases of recurrence were observed after surgical reexploration. Interestingly, a relapse was also seen in a case using a contained bag system.^19^ A recent study on 152 patients affected by early-stage uterine LMS also showed that any type of morcellation is related to risk of recurrence.^20^ Furthermore, in a retrospective multicenter study on 125 patients affected by uterine sarcomas or STUMPs, women undergoing either morcellation or power morcellation experienced a three-fold increase in the risk of death in comparison with patients who had not (*p* = 0.02), and a trend toward an increase of risk for recurrence was specifically found in the limited series (*n* = 11) of women undergoing morcellation for STUMP (HR 7.7, *p* = 0.09).^21^ The Food and Drug Administration (FDA)^22^ and the American College of Obstetricians and Gynecologists (ACOG)^23^ stated that morcellation should be avoided in patients over the age of 50 years, while for younger patients the possibility of a minimally invasive surgery should be carefully considered weighing the risk–benefit ratio. In this context, in-bag morcellation could reduce or nullify the risk of recurrence; however, solid evidence is lacking.^24^

Our data support that morcellation/fragmentation should not be performed if a STUMP is suspected and, if performed, close follow-up is advised.

### Histopathological Features

Histopathological classification of STUMPs represents a diagnostic gray area between leiomyomas and LMSs. To tackle this unmet diagnostic need, we evaluated the prognostic significance of several histological features potentially related to more aggressive tumor biology. The presence of epithelioid features and a higher mitotic count were found to be significant predictors of recurrence, while cellular atypia, which is included in STUMP diagnostic criteria,^14^ was not, similarly to other authors’ findings.^1,9–11,25^ STUMPs frequently show high cellularity; however, this finding also lacks correlation with outcomes^1,9,25^ as observed in our study. The presence of necrosis is a common feature of LMS;^26^ however, it can also be detected in STUMPs partly owing to tissue ischemia.^14^ Nevertheless, no relationship between necrosis and recurrence rates was conclusively demonstrated in previous studies,^1,8–10^ as well as in the present series. Similarly, the presence of apoptosis^1,8^ does not appear to be correlated with the risk of relapse.

Gupta et al.^8^ suggested that atypical mitoses, epithelioid differentiation, and vascular intrusion are possible predictors of recurrence. In the present series, we found higher recurrence rates and shorter RFSs in cases with epithelioid differentiation. In line with this observation, this feature was found to be related to more aggressive biological behaviors in studies analyzing the whole USMT spectrum.^27,28^ Myxoid features, another rare morphological trait that can mimic the inflammatory myofibroblastic tumor,^29^ did not correlate with worse outcomes.

Proliferation activity and mitotic count represent well-recognized prognostic markers of most soft tissue tumors;^30^ however, their significance in STUMPs is another controversial issue. In two previous studies, the mitotic count did not result as a significant prognostic parameter,^9,11^ while Ip et al.^1^ observed a trend toward lower mitotic counts in nonrecurring cases. Huo et al.^10^ proposed a cutoff of > 10 mitoses per 10 HPFs as a risk factor for recurrence, a finding confirmed by our study on multivariate analysis (although no association with RFS was observed). Finally, it should be noted that, in USMTs, the mitotic count has to be considered together with other histopathological features to correctly distinguish STUMPs from mitotically active leiomyomas and LMSs.^14^

Ki-67 expression is routinely evaluated in many neoplasms as a measure of proliferation activity, and in some cases, such as human epidermal growth factor receptor 2 (HER2)-negative luminal breast cancers, it can be used to identify which patients shall be treated with adjuvant chemotherapy.^31–33^ However, the usefulness of Ki-67 for USMTs is controversial. O’Neill et al.^17^ compared Ki-67 expression between 22 LMS and 41 other USMTs: 19 of 22 LMS showed a Ki-67 proliferation index > 20%, while all other USMTs had a Ki-67 proliferation index < 20%; however, the sample size of STUMPs in this study was remarkably low (only 4 cases). Mayerhofer et al.^34^ compared Ki-67 rates of 25 leiomyomas, 22 STUMPs, and 20 LMS: no STUMP showed an elevated Ki-67 expression; however, no recurrence was reported among these cases. Although methodological differences are present between the different studies (e.g., antibody used and counting method), available data suggest a higher expression of Ki-67 in LMS as expected; however, STUMPs are poorly represented and Ki-67 usefulness to predict their recurrence has been poorly explored so far. A recent meta-analysis^35^ suggested that Ki-67 is not useful to predict STUMPs recurrence; however, only 5 monocentric cohorts were analyzed in this study with a total sample size of 107 STUMPs and 15 recurrences only.^1,4,10,12,25^ Moreover, the authors provided data regarding a single Ki-67 cutoff value (10%), while our results suggest that a cutoff value of 20% is related to both recurrence and shorter RFS at univariate analysis.

The impact of hormone receptors (ER and PR) has been extensively studied in breast cancer and in different gynecological tumors, observing an association between their higher expression and better prognosis.^12,36,37^ In the present study, we detected, through univariate analysis, an association between lower expression of PR and a higher risk of relapse. Interestingly, a lower expression of PR is associated with worse prognosis in stage I LMS.^38^

Finally, we studied the potential role of p16 and p53 expressions. Alterations of these oncosuppressor proteins have been documented in STUMPs and LMSs.^39^ Some authors also suggested a predictive role, in terms of disease recurrence, of both p16 and p53 immunohistochemical expression,^1,10^ a finding confirmed by a recent systematic review and meta-analysis of literature.^39^ Nevertheless, the experience with these markers is still limited, and their evaluation is not recommended in clinical practice.^16^ We also found a potential relationship between diffuse p16 expression and risk of recurrence.

More recently, approaches based on molecular profiling have been proposed to improve STUMP stratification into benign and malignant lesions. Croce et al.^40^ analyzed the genomic profile of a series of different USMTs (24 STUMPs, 10 LMSs, and 10 leiomyomas) by array comparative genomic hybridization to evaluate the prognostic significance of genomic alterations (i.e., genomic index). These authors found that a specific genomic index threshold (index = 10) divides the STUMP category into two groups of neoplasms with different outcomes: a group comparable with leiomyomas and another similar to LMS, but with more indolent behavior. The same authors^41^ suggest that USMTs classified as stage I molecular LMS with 13q loss including RB1 and 17p gain including MYOCD gain are characterized by a worse prognosis. In another study,^42^ an array CGH analysis was performed on 23 USMTs (14 STUMPs, 5 LMSs, 3 leiomyomas, and 1 undifferentiated sarcoma), suggesting that the *PRKDC* and *PUM2* genes may have a prognostic role.

However, the number of STUMPs analyzed within these studies is relatively low,^40–42^ thus these findings should be validated/standardized on larger case series, and it should be noted that, to date, these approaches are not considered as diagnostic criteria according to the most recent diagnostic WHO classification.^14^

### Survival Outcomes

In this study, we reported a RFS and a DSS for STUMPs of 79.3% and 94.3%, respectively. A recent review^43^ reported a lower recurrence rate range (8.7–11%), similar to those reported by Gadducci et al.^16^ These values are lower compared with our study, but Gupta et al.^8^ reported a significantly higher recurrence rate (36.4%) in a series of 22 STUMPs, suggesting that some variations are present among neoplasms presently classified as STUMPs according to current diagnostic criteria. With our results, we propose new histopathological parameters that could be useful for discriminating high-risk from low-risk STUMPs; however, they remain a clearly distinct entity compared with LMS since the latter show significantly higher recurrence rates (45–75%)^44^ and poor 5-year survival rates (25–75%)^44^ compared with STUMPs (92–100%).^16^

### Strength and Limitations

The main limitations of this study are related to its retrospective nature, and to the potential differences in terms of patients’ management due to the lack of guidelines. Nevertheless, this study is, to the best of the authors’ knowledge, the largest multicenter series investigating STUMPs recurrence, and all samples and data were obtained and reviewed within tertiary referral centers with expert pathologists and clinicians.

## Conclusions

Focusing on surgical management, our results stress the importance of avoiding any type of morcellation in case of preoperative suspicion of STUMP and recommend careful follow-up in this subset of patients, while the surgical procedure and approach do not appear to be related to greater risk of recurrence. Pathological characteristics, epithelioid features, high proliferation activity, low PR expression, and diffuse p16 expression resulted associated with disease recurrence and shorter RFS.

Our data support the current classification of STUMPs as an entity different from classic LMSs. The identified variables can help distinguish a subset of cases with higher risk of recurrence that could represent a subgroup of low-grade LMS.^6^
