# Correction: Clinical and Histopathological Predictors of Recurrence in Uterine Smooth Muscle Tumor of Uncertain Malignant Potential (STUMP): A Multicenter Retrospective Cohort Study of Tertiary Centers

**DOI:** 10.1245/s10434-022-12594-x

**Published:** 2022-09-21

**Authors:** Fulvio Borella, Stefano Cosma, Domenico Ferraioli, Isabelle Ray-Coquard, Nicolas Chopin, Pierre Meeus, Vincent Cockenpot, Giorgio Valabrega, Giulia Scotto, Margherita Turinetto, Nicoletta Biglia, Luca Fuso, Luca Mariani, Dorella Franchi, Ailyn Mariela Vidal Urbinati, Ida Pino, Gianluca Bertschy, Mario Preti, Chiara Benedetto, Isabella Castellano, Paola Cassoni, Luca Bertero

**Affiliations:** 1 Division of Gynecology and Obstetrics 1, Department of Surgical Sciences, City of Health and Science University Hospital, University of Turin, Turin, Italy; 2 Léon Bérard Comprehensive Cancer Center, Lyon, France; 3 Department of Oncology, University of Turin, Turin, Italy; 4 Division of Obstetrics and Gynecology - A.O. Ordine Mauriziano, University of Turin, Turin, Italy; 5 Preventive Gynecology Unit, European Institute of Oncology IRCCS, Milan, Italy; 6 Pathology Unit, Department of Medical Sciences, University and City of Health and Science, Turin, Italy

## Correction to: Annals of Surgical Oncology https://doi.org/10.1245/s10434-022-12353-y

In the original online version of this article Giorgio Valabrega's family name was incorrectly spelled. The original aritcle was corrected.

